# Laparoscopic Colorectal Surgery in the Era of Robotics: Evolution, Eclipse, or Equilibrium?

**DOI:** 10.1002/ags3.70128

**Published:** 2025-11-19

**Authors:** Amanjeet Singh, Archit Gupta, Azhar Perwaiz, Adarsh Chaudhary

**Affiliations:** ^1^ Department of GI Surgery and GI Oncology Medanta the Medicity Gurugram India

**Keywords:** colorectal cancer, cost‐effectiveness, laparoscopic surgery, minimally invasive surgery, outcomes, robotic surgery

## Abstract

Minimally invasive colorectal surgery has undergone a remarkable transformation over the past three decades. Laparoscopy, once viewed with skepticism, is now firmly established as a standard approach, supported by robust randomized trials demonstrating oncologic safety and improved recovery compared to open surgery. More recently, robotic platforms have emerged, promising enhanced dexterity, three‐dimensional visualization, and ergonomic benefits. Their adoption has been rapid, with projections suggesting robotic resections may soon surpass laparoscopic procedures in some regions. Despite this momentum, evidence indicates that robotic surgery offers equivalence rather than superiority in most domains. Oncologic outcomes, postoperative recovery, and quality‐of‐life measures remain comparable between the two modalities. Robotics may provide advantages in technically demanding settings such as a narrow pelvis, obese patients, or after pelvic radiation, and some studies suggest improved urinary and sexual function. However, these benefits come at the cost of longer operative times and significantly higher expenses, raising concerns about accessibility and value, especially in resource‐constrained healthcare systems. Laparoscopy continues to offer broad global availability, faster setup, and cost‐effectiveness, while serving as the bedrock skill set for robotic mastery. The future of colorectal surgery will likely be defined not by one technology replacing another, but by their integration—augmented further by emerging innovations such as artificial intelligence, augmented reality, and next‐generation robotic systems. The challenge for surgeons and institutions is to apply these tools thoughtfully, ensuring that innovation is balanced with equity, sustainability, and clinical relevance.

## 1 Introduction

The story of surgery has always been one of precision, passion, and progress and nowhere is this more evident than in the evolution of minimally invasive colorectal surgery. Over the past three decades, laparoscopic techniques have transformed colorectal procedures by reducing morbidity, shortening hospital stays, and improving postoperative recovery [1, 2]. Once considered revolutionary, laparoscopy has become the standard of care in many colorectal operations worldwide.

However, just as the surgical world was getting comfortable with this paradigm, a new player entered the field: robotic surgery. With its promise of enhanced dexterity, 3D visualization, tremor filtration, and ergonomically favorable console‐based operation, robotic surgery has quickly captured the imagination of hospitals and surgeons alike [3, 4]. The rise of robotics in colorectal surgery has been meteoric, with large databases projecting that robotic‐assisted colectomies and proctectomies may surpass laparoscopy by 2026 in the United States [5].

Yet, as robotics ascends, laparoscopy is far from obsolete. It remains widely practiced, cost‐effective, and deeply embedded in surgical training and institutional protocols. While robotics may offer refinements, particularly in the narrow confines of the pelvis or for technically challenging procedures, the broader question remains: is robotic surgery truly replacing laparoscopy, or are we witnessing a recalibration of tools, where both coexist and complement each other?

This review aims to explore the current standing of laparoscopic colorectal surgery in the context of the robotic revolution. By critically examining the literature, surgical trends, clinical outcomes, cost implications, and training paradigms, we aim to provide clarity on whether robotics represents an evolution, an eclipse, or simply an expensive cousin in the family of minimally invasive surgery.

## 2 Historical Evolution and Current Role of Laparoscopic Colorectal Surgery

The birth of laparoscopic colorectal surgery was both revolutionary and controversial. While laparoscopy had made significant inroads in general surgery by the early 1990s, its application to colorectal procedures was initially met with scepticism. Concerns regarding oncologic safety, port‐site recurrences, and technical feasibility slowed its acceptance [6]. Yet, through persistence and innovation, laparoscopic colectomy evolved from an experimental technique to a widely endorsed standard.

Pivotal randomized trials such as the Clinical Outcomes of Surgical Therapy (COST), CLASICC, COLOR, and COREAN trials played an instrumental role in validating laparoscopic colorectal resection for cancer. These studies consistently demonstrated that laparoscopic approaches were non‐inferior to open surgery in terms of oncologic outcomes, such as lymph node harvest, margin status, and long‐term survival, while offering advantages in postoperative pain, bowel function, hospital stay, and cosmetic results (Table 1).

**TABLE 1:** Landmark trials establishing laparoscopy in colorectal surgery.

Trial	Year	*N*	Comparator	Main findings	Take‐home message
COST	2004	872	Open vs. Laparoscopy	Similar oncologic outcomes; shorter recovery with laparoscopy	Established safety in colon cancer
CLASICC	2005	794	Open vs. Laparoscopy	Higher conversion in lap group but equivalent margins	Proved feasibility in rectal cancer
COLOR II	2013	1044	Open vs. Laparoscopy (rectal)	Non‐inferior oncologic outcomes	Validated laparoscopy in rectal surgery
COREAN	2010	340	Open vs. Laparoscopy (rectal, post‐CRT)	Non‐inferior; faster recovery	Confirmed laparoscopy after neoadjuvant therapy

In colon cancer, laparoscopic surgery has gained broad acceptance and is often considered the default approach in many centers. In rectal cancer, the adoption has been more cautious, reflecting the complex pelvic anatomy, higher technical demands, and a need for precise total mesorectal excision (TME). Despite these challenges, data from trials such as COLOR II and COREAN demonstrated that experienced surgeons could achieve comparable outcomes laparoscopically in rectal cancer as well [1, 2, 7, 8, 9].

Beyond oncologic indications, laparoscopic colorectal surgery has expanded its utility to benign diseases, including diverticulitis, inflammatory bowel disease, and functional disorders. The minimally invasive approach has shown favorable outcomes in these settings, with reduced adhesions, faster recovery, and fewer wound complications [10, 11].

However, the journey hasn't been without hurdles. The steep learning curve for laparoscopic colorectal surgery, especially for rectal procedures, remains a significant barrier. Studies suggest that achieving proficiency in laparoscopic TME may require 50–100 cases or more [12, 13]. Pelvic dissection in obese males, narrow pelvis, bulky tumors, or prior radiation therapy adds to the complexity. As a result, laparoscopic rectal resection is still underutilized in some regions and often converted to open surgery in difficult cases [14].

Despite these challenges, laparoscopy has held its ground and even flourished in many settings. Its widespread adoption is supported by several pragmatic advantages. First, laparoscopy enjoys broad familiarity among general and colorectal surgeons due to well‐established training pathways. Second, it is cost‐effective, with substantially lower capital and maintenance expenses than robotic systems. Third, in straightforward cases, it often leads to shorter setup and operative times. Finally, many institutions already have perioperative care pathways optimized for laparoscopy, allowing for efficient implementation and patient management.

Data from the American College of Surgeons National Surgical Quality Improvement Program (ACS NSQIP) through 2023 continue to affirm laparoscopy's relevance. It still accounted for 43.5% of colectomies and 22.5% of proctectomies in the United States, maintaining a significant share of minimally invasive procedures even amidst the rising tide of robotic surgery [5].

Moreover, laparoscopic skills serve as the bedrock of robotic surgery. Robotic techniques build upon laparoscopic fundamentals—port placement, intra‐abdominal orientation, dissection planes, and management of complications. In many centers, surgical trainees are required to demonstrate laparoscopic competence before progressing to robotic platforms [15]. This foundational role ensures that laparoscopy is not just relevant, it's essential.

## 3 Rise of Robotic Colorectal Surgery

The surgical robot made its debut like a scene out of science fiction—sleek arms, intuitive consoles, and the tantalizing promise of precision beyond the limitations of the human hand. Since the FDA approved the da Vinci Surgical System in 2000, robotic surgery has steadily expanded its footprint, particularly in urologic and gynecologic procedures. However, its embrace in colorectal surgery has been more recent and deliberate, shaped by the pursuit of better ergonomics, improved visualization, and enhanced dexterity, especially in the anatomically constrained pelvis [3, 4].

Unlike conventional laparoscopy, where two‐dimensional vision and rigid instruments demand steep learning curves and considerable physical strain, robotic platforms offer 3D, high‐definition magnified views, and wristed instruments that replicate natural hand movements with seven degrees of freedom [16]. These features are particularly beneficial in rectal surgeries, where navigating the narrow male pelvis or irradiated fields often challenges even the most skilled laparoscopic surgeons [17].

Proponents of robotics cite its ergonomic superiority for the surgeon, translating into reduced physical fatigue during lengthy procedures. The console‐based approach allows for seated operation, better posture, and reduced musculoskeletal strain, which may improve surgeon longevity and focus [18]. Studies have also highlighted better nerve preservation, reduced conversion rates, and lower intraoperative blood loss in certain scenarios, especially for rectal cancer [19].

Yet, these benefits come with caveats. Robotic systems are expensive, not just in terms of initial capital cost, but also with maintenance contracts, disposable instruments, and longer operative times, particularly in the early phases of adoption [20, 21].

For legacy robotic systems, the loss of tactile feedback (haptics) was a longstanding concern, as laparoscopic surgery provides subtle resistance and tissue tension cues that early robotic consoles could not replicate. With the introduction of the da Vinci 5 platform, force feedback has been incorporated into selected instruments (notably the needle driver). However, this is not available across all tools, and use is contraindicated in certain tissue‐handling scenarios. Thus, while haptic limitations are being addressed, their integration remains partial and evolving [22, 23].

From a systems perspective, robotic surgery requires significant restructuring of the operating room environment. The presence of bulky robotic arms, vision carts, and consoles demands more space and precise choreography among the surgical team. Assistant ergonomics, often overlooked, can be poor—especially when navigating around robotic arms in constrained spaces.

According to ACS NSQIP registry data (2012–2023, U.S. colectomy and proctectomy codes), robotic colectomy utilization increased from < 1% to 24.3%, while proctectomy rose from 13.4% to 31.6%. A retrospective projection model (Violante et al. *Annals of Surgery* 2025, U.S. context) forecasts that by 2026 robotic colectomy will exceed laparoscopy (44.5% vs. 25.8%) and robotic proctectomy will similarly surpass laparoscopic procedures (38.3% vs. 20.1%). These figures should be interpreted as projections based on retrospective trend modeling, not confirmed registry outcomes [5]. These trends reflect both a shift in institutional investment and a growing confidence among colorectal surgeons in robotic platforms.

Yet, it remains unclear whether these shifts are driven by clinical superiority or institutional enthusiasm for high‐tech branding. Comparative studies and meta‐analyses have largely shown that while robotic colorectal surgery is feasible and safe, it does not offer a significant oncologic or functional advantage over laparoscopy in most cases [24]. In fact, the ROLARR trial, a large multicenter randomized controlled trial, found no significant difference in conversion rates between robotic and laparoscopic rectal surgery overall [25].

As the robotic landscape evolves, next‐generation systems such as CMR Surgical's Versius, Medtronic's Hugo RAS, and SSI Mantra are entering the market. These platforms aim to address prior limitations by offering modularity, compact design, better costing and greater access flexibility potentially expanding robotic adoption to smaller hospitals and broader geographic regions.

Despite these limitations, the field is evolving. Next‐generation platforms are on the horizon, promising smaller footprints, modular designs, improved haptics, and even AI‐assisted surgery. The future may also include telesurgery, remote mentoring, and semi‐autonomous robotic actions, opening doors previously unimaginable.

For now, robotic colorectal surgery represents both progress and paradox—an elegant solution that may not yet justify its cost in every scenario, but one that continues to push the boundaries of what is surgically possible.

## 4 Comparative Outcomes: Robotic vs. Laparoscopic Colorectal Surgery

The conversation around robotic versus laparoscopic colorectal surgery is not just about technology; it is about what truly improves patient outcomes. While robotics dazzles with precision, laparoscopy boasts decades of proven performance. Comparing the two requires a careful look at clinical data, beyond the shimmer of steel and screens.

### 4.1 Operative Time and Learning Curve

Robotic colorectal surgery is frequently associated with longer operative times, especially during the early phases of adoption. Studies like the ROLARR trial demonstrated a significantly longer mean operative time for robotic procedures (mean difference of approximately 37 min) compared to laparoscopy [25]. However, with increasing experience and improved system familiarity, operative times tend to decrease and may even match laparoscopy in high‐volume centers.

Interestingly, the learning curve for robotic rectal surgery may be shorter than that of laparoscopy. Some studies suggest proficiency in robotic total mesorectal excision (TME) may be achieved in as few as 20–40 cases, compared to 50–100 cases for laparoscopy [26]. This is particularly relevant for pelvic procedures where robotic articulation and stable visualization offer tangible ergonomic advantages.

### 4.2 Conversion Rates

Conversion to open surgery remains a critical outcome metric in minimally invasive colorectal procedures. One of the most cited advantages of robotic surgery has been its potentially lower conversion rates, especially in difficult pelvic dissections. However, data remains mixed. The ROLARR randomized controlled trial concluded that robotic rectal resection did not significantly reduce the risk of conversion to open surgery compared with laparoscopy (8.1% vs. 12.2%, *p* = 0.16), and overall showed no superiority of robotics over conventional laparoscopy [25].

Subgroup analyses in obese patients and males with narrow pelvis did suggest a trend toward lower conversion rates with robotics, although these findings did not reach statistical significance. Other retrospective studies and meta‐analyses have echoed similar results, with marginally lower conversion rates seen in robotic groups but without robust superiority [19, 24].

### 4.3 Postoperative Outcomes

In terms of blood loss, the robotic approach tends to have a slight edge, with studies consistently reporting reduced intraoperative bleeding compared to laparoscopy [27]. However, this difference is usually modest and may not significantly impact overall morbidity.

Postoperative recovery, including metrics like time to bowel function, length of stay, and pain scores, tends to be comparable between the two approaches. Robotic surgery has not demonstrated clear superiority in these parameters, and in many institutions, outcomes appear to be more related to enhanced recovery protocols (ERAS) than the surgical modality itself.

### 4.4 Oncologic Outcomes

Perhaps the most important question in cancer surgery is whether robotic surgery offers better oncologic outcomes. Here, laparoscopy has long‐established credibility, and robotic surgery has, at best, matched it. Trials such as COLOR II, COREAN, and CLASICC affirmed the safety of laparoscopic resection in colorectal cancer [2, 7, 28].

Robotic surgery has demonstrated comparable results in terms of R0 resection rates, lymph node yield, and circumferential resection margin positivity. No randomized controlled trial to date has shown superior long‐term survival or disease‐free survival with robotic surgery over laparoscopy. In rectal cancer, where precise TME is crucial, robotic systems offer technical advantages but have not translated into a clear oncologic benefit in outcome studies.

While most large trials originate from Western countries, emerging data from Asia adds regional relevance. Studies from high‐volume centers in India, South Korea, and Japan have demonstrated feasibility and favorable outcomes with robotic rectal surgery, even in lower‐resource settings. Furthermore, ongoing trials such as the RECOLORS Study in Japan and the ROBO‐Colon Trial in India aim to evaluate long‐term outcomes and cost‐effectiveness in Asian populations, offering a more geographically inclusive evidence base.

### 4.5 Functional and Quality‐Of‐Life Outcomes

Preservation of urinary and sexual function, particularly in rectal surgery, is an area where robotics has shown potential advantages. Robotic instruments allow finer dissection near the pelvic autonomic nerves, theoretically reducing iatrogenic injury. Some small studies and non‐randomized trials have reported better functional outcomes in this domain, but evidence remains limited and heterogeneous [29].

Quality‐of‐life measures, including body image and return to baseline activity, appear similar between robotic and laparoscopic approaches when assessed longitudinally. Patient satisfaction may be slightly higher with robotic surgery due to perceptions of advanced technology, though this is not consistently reflected in objective data.

### 4.6 Cost‐Effectiveness

Robotic surgery is undeniably more expensive than laparoscopy. Higher capital investment, recurring maintenance, and consumables contribute to significantly increased costs. While some analyses suggest potential offset in select high‐risk populations (e.g., obese males, narrow pelvis), broader cost‐effectiveness remains controversial. In healthcare systems with constrained resources, laparoscopy maintains a decisive advantage (Table 2).

**TABLE 2:** Key comparative studies: Laparoscopic vs. robotic colorectal surgery.

Year	Study (design)	Population/*N*	Comparison	Main findings	Take‐home message
2009	Baik et al. [19] *Annals of Surgical Oncology* (Prospective cohort)	Rectal cancer, *N* = 100	Robotic vs. Lap TME	Similar oncologic outcomes; robotics ↓ conversion trend, improved nerve preservation	Robotics feasible, possible functional advantage
2012	Trastulli et al. [24] *Colorectal Disease* (Systematic review & meta‐analysis)	Pooled ~1200 pts	Robotic vs. Lap rectal resection	No significant oncologic benefit; longer OR time for robotics	Robotics safe, not superior
2014	Kim et al. [20] *Journal of Gastrointestinal Surgery* (Systematic review)	Multiple RCTs & cohorts	Robotic vs. Lap	Equivalent outcomes; ↑ operative time & cost in robotic	Robotics adds no clear clinical benefit
2014	Liao et al. [27] *World Journal of Surgical Oncology* (Meta‐analysis, 4 RCTs)	~400 pts	Robotic vs. Lap colorectal	Robotics ↓ blood loss; other outcomes similar	Marginal peri‐op advantage
2017	ROLARR Trial, *Journal of the American Medical Association* (RCT, *N* = 471) [25]	Rectal cancer	Robotic vs. Lap	No significant ↓ conversion rate (8.1% vs. 12.2%); longer OR time in robotic	Robotics not superior overall; subgroup benefit possible
2018	Kim et al. [29] *Colorectal Disease* (Propensity‐matched)	Rectal cancer, *N* = 300	Robotic vs. Lap	Better urinary/sexual recovery at 12 months in robotic group	Functional recovery may favor robotics
2022	Tang et al. [26] *Frontiers in Oncology* (Learning curve analysis)	Rectal cancer, Robotic TME	Learning curve analysis	Robotics proficiency at ~20–40 cases vs. 50–100 for lap	Robotics shortens learning curve
2023	Pietersen et al. [15] *Surgical Endoscopy* (Systematic review)	Multiple studies	Skill transfer Lap ↔ Robotics	Lap skills → Robotics transfer better than reverse	Lap remains essential foundation
2025	Guerrero‐Ortíz et al. [30] *Surgery* (ROBOCOSTES Trial)	Rectal cancer, Spain (National prospective trial)	Robotic vs. Lap	Better short‐term outcomes with robotics; cost ↑ €920 per case	Robotics feasible, but cost‐effectiveness borderline

## 5 Training and the Learning Curve in the Robotic Era

The transition from open to minimally invasive surgery has dramatically reshaped surgical education, but the robotic revolution has ushered in an even steeper transformation. While laparoscopy once represented the pinnacle of minimally invasive mastery, robotic platforms now demand an entirely new set of skills ranging from console navigation and docking protocols to camera control, wristed articulation, and teamwork choreography. As the adoption of robotics accelerates, surgical education is scrambling to keep pace.

Laparoscopic colorectal surgery, especially rectal procedures, is notoriously difficult to master. The learning curve is steep, with studies estimating that 50–100 cases are required to achieve consistent proficiency in oncologic dissection and complication management. The skill set includes precise two‐handed coordination, depth perception from a 2D screen, and counterintuitive instrument movements, all performed while standing for hours, often in ergonomically challenging positions.

Robotic systems were, in part, developed to address these very hurdles. By offering three‐dimensional visualization, wristed instruments with seven degrees of freedom, and intuitive motion control, robotic platforms shorten the cognitive load required for technical maneuvers. Multiple studies suggest that proficiency in robotic total mesorectal excision (TME) can be achieved in as few as 20–40 cases. This is particularly significant in pelvic surgery, where the robotic interface simplifies delicate nerve‐sparing dissections in confined spaces.

However, robotics does not eliminate the need for foundational laparoscopic skills. Port placement, abdominal access, and intraoperative complication management still rely on laparoscopic principles, especially for the patient‐side assistant who often manages critical portions of the case. Laparoscopy remains the gateway through which most surgical residents are introduced to MIS, and robotic surgery is typically built atop this platform.

This dual training requirement presents a challenge for residency and fellowship programs. Curricula must now accommodate both laparoscopic and robotic skill development, often without extending the total duration of training. Several institutions have integrated simulation‐based modules, dry‐lab training, and dual‐console teaching models to scaffold robotic education. Structured programs such as the Fundamentals of Robotic Surgery (FRS) and the European Academy of Robotic Colorectal Surgery curriculum offer competency‐based learning pathways, but access to such programs varies widely [31].

A systematic review by Pietersen et al. in 2023 found that while laparoscopic skills were moderately transferable to robotic surgery, the reverse was less consistent, highlighting that laparoscopic training remains essential for comprehensive MIS education [15]. This suggests that abandoning laparoscopy entirely in favor of robotics could create gaps in surgical competence, particularly in institutions where robotic platforms are not always available.

Moreover, robotic surgery shifts the dynamics of team training. Unlike laparoscopy, where the primary surgeon and assistant often stand side by side, robotics creates physical separation. This necessitates dedicated training not only for the console surgeon, but also for bedside assistants, scrub nurses, and anesthesiologists, who must coordinate with a remote operator. Proctoring, credentialing, and formal robotic case logs are now required in many institutions before a surgeon can perform independent robotic cases, further emphasizing the complexity of integration.

Importantly, the training infrastructure remains unequal globally. In low‐ and middle‐income countries, laparoscopy is often the only feasible MIS option due to cost and access limitations. For these regions, robust laparoscopic training remains not only relevant but vital.

Training disparities persist between urban tertiary centers and rural surgical facilities. While academic institutions increasingly incorporate robotic curricula and simulation labs, many smaller centers, especially in LMICs lack access to robotic platforms or certified proctors. Bridging this gap will require scalable training models and cross‐institutional partnerships to democratize robotic education.

In summary, robotic surgery may flatten certain technical learning curves, but it introduces new ones in team coordination, simulation‐based credentialing, and institutional workflow. Rather than replacing laparoscopic training, it demands a reimagined approach to surgical education, one that preserves the strengths of laparoscopy while building competency in a robotic future.

## 6 Economics and Accessibility: Cost, Resource Allocation, and Equity

In the glistening world of robotic surgery—complete with console control, wristed articulation, and 3D vision—there exists a less glamorous, but crucial variable: Cost. For many institutions, particularly in resource‐constrained settings, economic feasibility determines whether robotics becomes a reality or remains a showroom fantasy.

Robotic colorectal surgery is significantly more expensive than laparoscopic surgery, a fact supported consistently across cost‐analysis studies. Capital investment in a robotic platform ranges between $1.5 and $2.5 million, with annual maintenance costs approximating $100 000–$200 000. Each case adds further expense through proprietary robotic instruments, disposable arms, and vision system supplies, often pushing the per‐case cost 1.3 to 2 times higher than laparoscopy [32].

Even in high‐income countries, cost‐effectiveness analyses have raised questions. Keller et al. showed that although robotic surgery offered marginal improvements in complication rates and patient satisfaction, these did not always translate into meaningful cost savings when compared to laparoscopic procedures. Robotic proctectomy, in particular, may approach cost neutrality in high‐risk or obese patients, where reduced conversions and better access in the pelvis can offset the increased equipment expenditure [33].

Cost‐effectiveness is even more starkly skewed in low‐ and middle‐income countries (LMICs), where the capital cost of a robotic platform may equal the annual budget of an entire surgical department. For such systems, laparoscopy remains the only viable form of minimally invasive colorectal surgery. This creates an equity gap: as high‐income regions increasingly shift toward robotic MIS, patients in resource‐limited areas may face a growing technology divide, with limited access to what is perceived as state‐of‐the‐art care.

Insurance coverage also plays a role in shaping accessibility. In countries with universal healthcare, such as the UK and Canada, robotic surgery may be selectively covered for certain indications, while others remain restricted due to cost containment measures. In contrast, countries like the U.S. and South Korea have seen broader but uneven adoption, often dependent on private hospital systems and patient affordability [21, 34].

This disparity has sparked debate about the value of robotic surgery in routine cases. A recent multicenter European cost‐effectiveness analysis comparing robotic and laparoscopic rectal resections found robotic surgery was associated with better short‐term outcomes but at an additional cost of €920 per case, offset partially by improved QALYs [30]. Unless robotic platforms demonstrate significant outcome advantages in complex or high‐risk cases, their cost‐effectiveness remains questionable in routine colorectal surgery.

Additionally, the bulk and complexity of robotic platforms require dedicated infrastructure—larger operating rooms, specialized support staff, and increased turnaround time. These “hidden costs” may not be immediately reflected in billing statements, but they influence scheduling, resource allocation, and training time (Table 3).

**TABLE 3:** Economic and accessibility comparison: Robotic vs. laparoscopic colorectal surgery.

Parameter	Robotic surgery	Laparoscopic surgery	Notes/implications
Capital cost	$1.5 M–$2.5 M (initial system cost)	$100 K–$300 K (laparoscopic towers and instruments)	Major barrier to entry for smaller centers and LMICs
Maintenance cost	$100 K–$200 K/year	Minimal (non‐proprietary instruments)	Ongoing burden; robotic systems require service contracts
Per‐case instrument cost	$800–$2000 (disposables, robotic arms)	$100–$500 (reusable instruments, staples)	Proprietary single‐use tools increase robotic cost per case
Operating room time	Longer setup and docking; total OR time 15–40 min higher in early adoption phase	Shorter operative time in experienced hands	May even out with volume and team experience
Staff and infrastructure needs	Specialized staff, console, vision cart, larger OR space	Standard MIS team	Robotic workflow demands space and team reorganization
Insurance/reimbursement	Often covered in high‐income settings; limited or self‐pay in LMICs	Widely reimbursed, standard of care	Disparity in patient access and institutional support
Cost‐effectiveness	Justifiable in high‐risk pelvic cases; not cost‐effective for routine resections	Proven cost‐effectiveness across indications	Robotic value higher in obese, narrow pelvis, irradiated field
Global accessibility	Concentrated in urban academic centers and private hospitals	Available in both public and private sectors, globally	Significant equity gap between high‐income and low‐income healthcare systems

Abbreviations: LMICs = low‐ and middle‐income countries, OR = operating room.

That said, the robotics market is evolving. Emerging platforms from new manufacturers are introducing competitive pricing, modular designs, and system interoperability. These developments, alongside increasing global demand, may help drive down costs. Time‐driven activity‐based costing models have shown that with increased case volume, robotic surgery can approach cost neutrality, particularly in high‐throughput academic centers.

Ultimately, the economics of robotic surgery are nuanced. It may not be cost‐effective for all patients or institutions, but it offers potential value in select complex cases, particularly where surgical access is difficult or conversion risk is high. Laparoscopy, with its proven outcomes and lower cost, continues to be the cornerstone of MIS in colorectal surgery, particularly in settings where value‐based care is paramount.

Adoption trends vary significantly across nations. Japan has seen rapid integration of robotic colorectal surgery, supported by staged national insurance reimbursement milestones, initially for rectal procedures in April 2018, and later for colon procedures in April 2022. These milestones, together with evolving national guidelines, have facilitated wider adoption [35]. In India, uptake remains limited to private and metropolitan centers due to high costs and limited infrastructure, though public–private partnerships and training fellowships are slowly emerging to improve access and skill dissemination.

## 7 Environmental Sustainability: The Silent Cost of Innovation

While robotic surgery often earns applause for its innovation and precision, it comes with an environmental price tag that is rarely part of the operating room checklist. As surgical systems grow more complex, so too does their carbon footprint—and the consequences extend beyond hospital walls.

Several studies have highlighted the significantly higher environmental burden of robotic surgery compared to laparoscopic techniques. A systematic review by Papadopoulou et al. (*British Journal of Surgery* 2022) synthesized available environmental studies and reported that robotic colorectal procedures generated approximately 43.5% more greenhouse gas emissions and 24% more surgical waste than laparoscopic approaches. The authors emphasized, however, that these figures varied depending on the system boundaries applied in each analysis [36]. These differences stem from multiple sources: increased energy consumption by robotic consoles and vision systems, disposable robotic instruments with limited reusability, and larger sterile draping requirements.

Unlike laparoscopic tools, which are often reusable after sterilization, robotic instruments are frequently single‐use or have restricted reuse cycles, after which they must be discarded. Moreover, the extensive packaging and protective casings used for robotic arms and vision components contribute to excess medical waste, much of which is non‐biodegradable and difficult to recycle due to contamination risks.

Operating rooms already account for up to 30% of hospital waste and roughly 60% of all hospital GHG emissions. As robotic adoption expands, so does its environmental imprint, posing ethical challenges in sustainability‐conscious healthcare systems. Some institutions have begun implementing green OR initiatives, such as waste segregation, reprocessing protocols, and energy audits, but these efforts often lag behind the pace of robotic expansion.

Beyond simply describing the problem, it is important to highlight actionable strategies that can mitigate the environmental footprint of minimally invasive surgery. Prioritizing reusable instruments where safe and feasible, rationalizing instrument trays to avoid waste, and ensuring proper segregation and recycling of OR materials can substantially reduce disposable load. Energy stewardship, such as powering down consoles between cases and optimizing HVAC systems, together with low‐flow pneumoperitoneum and smoke evacuation, further contribute to greener operating practices. Anesthetic choices also matter, with the avoidance of high global‐warming potential agents such as desflurane and nitrous oxide when appropriate. Extending the validated life cycle of robotic instruments, incorporating sustainability criteria into procurement, and introducing feedback mechanisms such as “green OR” audits can embed accountability into practice. Ultimately, fostering a culture of environmental consciousness through education and designating “green champions” within surgical teams ensures that sustainability is not an afterthought but a standard part of innovation [36].

The environmental cost becomes even more relevant in regions already struggling with pollution and climate vulnerability. In low‐resource settings, where power supply is inconsistent and waste management infrastructure is limited, the introduction of high‐energy, high‐waste surgical systems may strain systems further, ironically widening the global gap in health equity under the guise of surgical modernization.

Surgical societies and manufacturers are increasingly being called upon to innovate not just for precision but also for planet‐conscious practice. Next‐generation platforms with modular reusable components, low‐energy modes, and biodegradable packaging could shift the balance. Until then, surgeons, administrators, and policymakers must weigh not just clinical outcomes and financial cost but also environmental consequences when choosing between laparoscopic and robotic techniques.

## 8 The Forgotten Player: Challenges for the Bedside Assistant

While much of the robotic surgery narrative centers around the console surgeon's experience, the role of the bedside assistant—often a junior surgeon or scrub assistant—is frequently underappreciated and underdiscussed. Ironically, as the surgeon moves away from the patient and into a console, the assistant steps into a more physically and technically demanding role.

In laparoscopic surgery, the assistant works in tandem with the primary surgeon, often sharing visual cues, tactile feedback, and coordinated movements. But in robotic surgery, the assistant is left managing critical intra‐abdominal steps—suctioning, retraction, stapler placement, clip application, often without the full operative view and with minimal guidance due to physical separation from the console operator.

The ergonomics of this role are poor. Assistants are frequently wedged between bulky robotic arms in awkward positions, with restricted access to the operative field. This can lead to frustration, longer operative times, and even increased risk of intraoperative collisions or injury. In emergencies, where undocking the robot is required, the assistant becomes the de facto lead until the console surgeon can re‐engage, posing both technical and psychological strain.

Furthermore, many training programs do not offer structured guidance for robotic assistants, even though their role is critical to procedural success. Unlike console training, which now includes simulations, dual‐console mentorship, and certification pathways, the assistant role remains largely informal and on‐the‐job—a major gap in current robotic surgical education.

For institutions adopting robotic surgery, investment in assistant training is not optional—it is essential. As robotic platforms evolve, greater emphasis on team training, shared mental models, and bidirectional communication between console and bedside must be prioritized.

## 9 Future Directions and Conclusions

As surgical innovation continues its unrelenting march forward, robotic colorectal surgery stands at the crossroads of promise and practicality. Its precision, stability, and ergonomic sophistication represent a natural evolution of minimally invasive techniques. Yet, it is not a panacea. Like all powerful tools, its value lies in thoughtful application, not universal adoption.

The future of colorectal surgery will likely be defined not by a single technology, but by integration, a seamless convergence of robotics, artificial intelligence, augmented reality, and data‐driven decision‐making. Emerging platforms such as Hugo, Versius, and SSI Mantra herald a new era of competition and affordability, potentially expanding robotic access to a wider range of institutions, including those in resource‐limited settings. In parallel, AI‐assisted skill assessment, autonomous suturing, and real‐time analytics are transforming the way we train, evaluate, and enhance surgical performance.

Despite the widespread use of the term “robotic,” it is essential to acknowledge that current systems primarily function as sophisticated laparoscopic manipulators. They operate through a master–slave configuration, replicating the surgeon's movements without any autonomous capability. Surgical outcomes remain highly dependent on the operating surgeon's expertise, and the technology, while advanced, does not yet offer true cognitive or decision‐making support.

Looking ahead, the next transformative leap will be driven by the integration of artificial intelligence, ushering in systems capable of real‐time anatomical recognition, adaptive responses, and partial autonomy. Such advancements hold the potential to minimize inter‐surgeon variability, standardize care, and democratize access to complex surgical procedures. The shift from robotic assistance to genuine collaboration, and eventually autonomy, is no longer hypothetical; it is on the horizon.

As the field evolves, so must our lens. Robotic platforms must demonstrate not only technological superiority but also clinical efficacy, cost‐effectiveness, environmental sustainability, and equitable accessibility. Institutions must be prepared to invest not just in machines, but in training, maintenance, and team integration, ensuring bedside assistants, rural surgeons, and public hospitals are not left behind in the shadow of gleaming robotic arms.

For the developing world, where cost constraints and infrastructure challenges persist, laparoscopic surgery remains an essential cornerstone—safe, effective, and accessible. The challenge, therefore, is not to replace laparoscopy, but to redefine when, where, and for whom robotic surgery adds real value. This calls for more region‐specific studies, pragmatic trials, and outcome registries that capture the lived realities of diverse surgical ecosystems.

While the trajectory of innovation is clear, significant gaps in evidence remain that must be addressed before robotics can fully justify its place alongside or above laparoscopy. Randomized controlled trials in obese patients, those with narrow male pelvises, or irradiated fields remain scarce, and region‐specific data from low‐ and middle‐income countries are urgently needed. Long‐term outcomes, particularly regarding urinary and sexual function after rectal surgery, are inconsistently reported and demand harmonized, prospective measurement. Cost‐effectiveness analyses should move beyond high‐income settings to reflect the realities of public hospitals and mixed insurance systems. Environmental studies require standardized life‐cycle assessments with clear boundaries to make comparisons meaningful. Training science must evolve to capture not just console proficiency but also the ergonomics and workload of bedside assistants and the broader surgical team. Finally, as artificial intelligence enters the arena, agreed metrics for phase recognition, skill assessment, and workflow efficiency must be defined, while large‐scale registries linking procedure data with oncologic, functional, and environmental outcomes will be critical to closing the evidence gap.

As surgeons, our compass must remain unwavering: to offer patients the best possible outcome using the most appropriate approach for their unique context. Robotic colorectal surgery is no longer a futuristic dream; it is a present‐day option. But whether it becomes the default path or a specialized lane depends on how responsibly and reflectively we wield it.

In the end, it is not about the machine; it is about the mission. Precision must walk hand in hand with purpose, and innovation must never outpace intention. Only then can we ensure that the future of colorectal surgery is not just advanced, but truly better for all.

## Author Contributions

**Amanjeet Singh:** conceptualization, investigation, writing – review and editing, visualization, validation, methodology, writing – original draft. **Archit Gupta:** conceptualization, investigation, writing – original draft, visualization, methodology, validation. **Azhar Perwaiz:** conceptualization, investigation, writing – review and editing, validation, visualization. **Adarsh Chaudhary:** conceptualization, investigation, writing – review and editing, visualization, validation, methodology.

## Conflicts of Interest

The authors declare no conflicts of interest.
